# Current progress in the inflammatory background of angiogenesis in gynecological cancers

**DOI:** 10.1007/s00011-019-01215-1

**Published:** 2019-01-24

**Authors:** Grzegorz Szewczyk, Tomasz M. Maciejewski, Dariusz Szukiewicz

**Affiliations:** 1 Chair and Department of General and Experimental Pathology, Medical University of Warsaw, ul. Pawinskiego 3C, 02-106 Warsaw, Poland; 2 Department of Gynecology and Obstetrics, Institute of Mother and Child, ul. Kasprzaka 17A, 01-211 Warsaw, Poland

**Keywords:** Angiogenesis, Cancer, Inflammation, Extracellular matrix

## Abstract

A tumor growth depends on the potency of the tumor to support itself with nutrients and oxygen. The development of a vascular network within the tumor is key to its survival. The permanent contest between the tumor and its host involves tumor cells on one side and an immunological system and tissue stroma on the other. The angiogenesis is not only a specialty of the tumor, but it also depends on this complex multidirectional interaction. The most common gynecological cancers, cervical, endometrial and ovarian carcinoma are good examples for studying this problem. In this review, we aim to show that an inflammatory response against a tumor can be reverted into an undesirable process leading to the development of a vascular network within the tumor and, subsequently, further growth of the tumor and progression of a disease. Therefore, a key for tumor management should be searched within the immunological system, rather than focused on cell cycle and anti-angiogenic treatment only.

## Introduction

Angiogenesis plays a crucial role in the pathogenesis of some gynecological diseases including endometriosis and malignant tumors. The formation of new blood vessels is achieved by a sprouting from preexisting vessels and requires proliferation and migration of endothelial cells (EC). Physiological angiogenesis is reserved for just a few particular events in human life: fetal development, menstruation cycle and wounds repair. The other aspect of angiogenesis (apart from physiology) is the participation in the pathogenesis of some disorders for which cellular growth is a common feature. The Folkman’s basic theory states that each tumor over 1–2 mm^3^ must gain access to vasculature for further growth [1]. The control of angiogenesis is achieved through the balance between both pro- and anti-angiogenic factors, with a dominant shift to proangiogenic in course of malignant diseases. Hypoxic conditions are a well-known stimulator of angiogenesis, mainly acting through hypoxia-inducible factor-1α (HIF-1α) which directly activates the expression of pro-angiogenic proteins like vascular endothelial growth factor (VEGF), vascular endothelial growth factor receptor-1 (VEGFR-1), tyrosine kinase with immunoglobulin-like and EGF-like domains 2 (TIE-2) receptor and many others [2]. There is a lot of proof that hypoxia is not alone, inflammation also plays a regulatory role in angiogenesis. Not surprisingly, as inflammation accompanies tumors in a primary site as well as in metastases [3]. Moreover, the inflammatory process influences EC, fibroblasts and other compounds of extracellular matrix (ECM) with the same molecular mechanisms as observed during angiogenesis [4]. The interaction between tumor cells, the inflammatory process and angiogenesis can overlap each other, thus it is not so easy to discuss them separately. In this paper, we will focus on the mechanisms which can be recognized as inflammation-associated angiogenesis in gynecological cancers.

## Immunocompetent cells in tumor vicinity

**Fig. 1:**
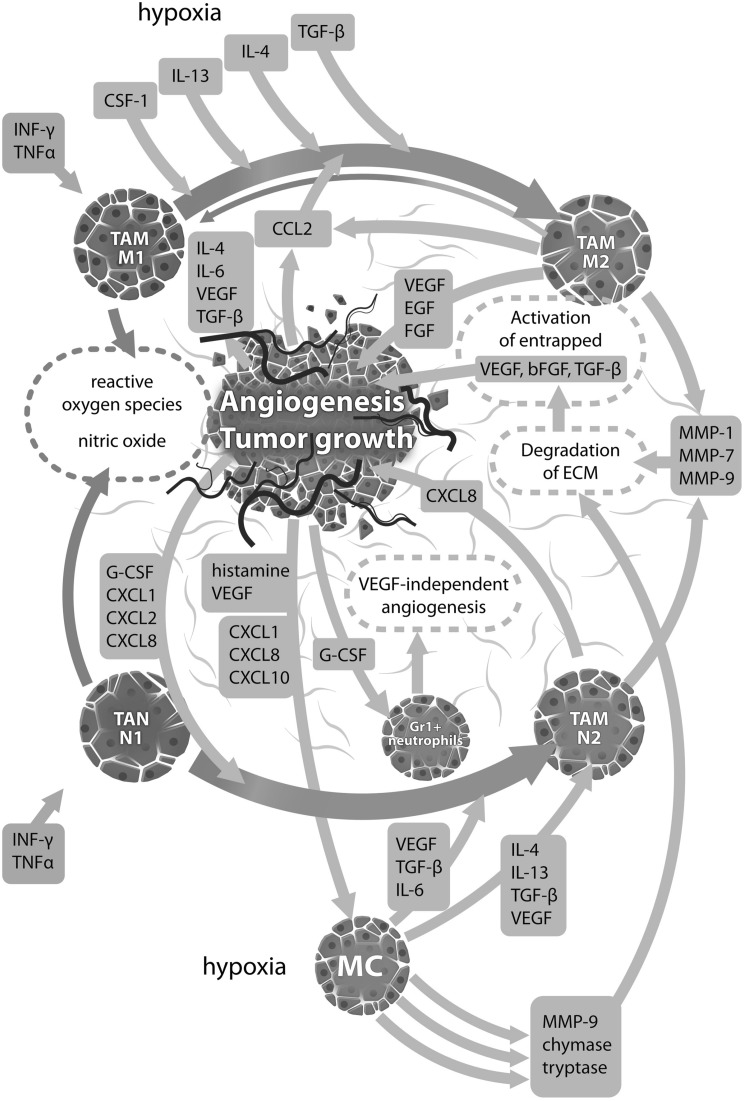
A complex interaction among inflammatory antitumor cells (TAM M1, TAN N1), their products, tumor surrounding including ECM and inflammatory protumor cells (mast cells, TAM M2, TAN N2). Cytokines, interleukins and growth factors are released in hypoxic environment of tumor from both tumor and immunocompetent cells, leading to angiogenesis and autocrine stimulation of macrophages and neutrophils. Angiogenesis is driven directly by the release of proangiogenic factors like VEGF and bFGF from inflammatory cells and indirectly by degradation of ECM and delivery of entrapped angiogenic chemokines or by stimulation of tumor cells to production of VEGF and other chemokines. Tumor cells produce chemokines that participate in a transformation of M1 and N1 cells to their “bad” analogues, conversely M2 and N2, stimulate mast cells to angiogenic activity and stimulate a specific type of neutrophils (Gr1+) to VEGF-independent angiogenesis. For further explanation see text. *TAM M1* tumor-associated macrophages type 1, *TAN N1* tumor-associated neutrophils type 1, *ECM* extracellular matrix, *TAM M2* tumor-associated macrophages type 2, *TAN N2* tumor-associated neutrophils type 2, *MC* mast cells. Graphic was made with the use of Affinity Designer

Phenotypic features of the tumor are one of the determinants defining the potential to growth. Nevertheless, the microenvironment must be “cooperative” to give a base for this growth. This cooperation is achieved through autocrine and paracrine stimulation of tumor cells together with neighbouring fibroblasts and inflammatory cells, thus creating a complex network of overlapping interactions within the tumor microenvironment (Fig. 1).

Numerous immune cells were found in the tumor surroundings. These include: macrophages, which seem to be dominant, then neutrophils and mast cells; while on the other side of “the barricade” there are lymphocytes—their presence seems to be a positive prognostic factor [5]. The comprehensive lists of immunocompetent cells and their function is given in Table 1 and broadly explained below.

**Table 1:** Different functions of immunocompetent cells in the facet of angiogenesis and their role in gynecological tumors

Cell type	Stimulator	Function	Tumor type and specific role	References
TAM M1	INF-γ TNF-α	Release of reactive oxygen species and nitric oxide to provide cytotoxic effect, release of IL-12, IL-23 to induce Th1 antitumor response	Ovarian cancer—accumulation improves prognosis; decreased angiogenesis, tumor regression	[6] [9] [26] [33, 34] [42]
TAM M2	TGF-β IL-4 IL-13 CCL2 Hypoxia CSF-1 Tumor cytokines Il-6, VEGF	Release of VEGF, EGF, and FGF to promote tumor growth and angiogenesis Synthesis of MMP-7 and MMP-9	Cervical cancer—increased angiogenesis, increased lymphangiogenesis, worse response to treatment and worse overall survival Endometrial cancer—correlation with loss of differentiation; increased angiogenesis, lower progression free survival Ovarian cancer—poor prognosis correlates with M2 stimulation	[10] [12] [19] [20] [21] [22] [23, 24] [27–29] [30] [31] [32] [43]
TAN	CXCL1, CXCL2 INF- γ, TNF-α G-CSF IL-8	Intracellular VEGF release MMP-9 synthesis Release of Bv8 Release of CXCL8	Cervical cancer—low recurrence-free survival correlates with accumulation of TANs Ovarian cancer—increased angiogenesis Endometrial cancer—increased tumor growth	[47, 49, 50] [53] [56] [59] [62] [61] [64]
CD11^+^Gr1^+^ neutrophils	G-CSF	Alternative to VEGF angiogenesis pathway	Animal models only	[55]
Mast cells	Hypoxia IgE Adenosine CRH	Release of Histamine, chymase, tryptase Leukotrienes, prostaglandins Cytokines: IL-1α, IL-1β, IL-3, IL-4, IL-5, IL-6, IL-9, IL- 10, IL-12, IL-13, IL-15, IL-16, IL-18, IFN-α, TGF-β, VEGF Autocrine stimulation with CCL2, CXCL1, CXCL10, PGE_2_, histamine, VEGF, Ang1, CXCL8/IL-8	Endometrial cancer—poor prognosis, loss of differentiation with accumulation of mast cells Cervical cancer—tumor progression, increased angiogenesis and invasiveness Ovarian cancer—better prognosis	[68] [70] [73] [75] [76] [77] [78] [80]
TILs-Treg FoxP3+	?	Positive correlation with vascular density	Cervical cancer—decreased overall survival Endometrial cancer—increased angiogenesis with no impact on survival	[89] [87]

*?* unknown

### Tumor-associated macrophages

Tumor-associated macrophages (TAM) are dominant immune cells present in a solid tumor, even totaling up to 50% of the volume [6]. The population of TAM can be heterogeneous and the probable reason are different levels of hypoxia in different sites of the tumor [7, 8]. Two major phenotypes of TAM are distinguished: M1 and M2. M1 represents the classically activated macrophages, which suppress tumor growth, while the M2 phenotype, with an alternative route of activation, is believed to play a stimulatory role in tumor development. M1 TAM are activated with interferon-γ (INF-γ) and tumor necrosis factor-α (TNF-α) to provide a cytotoxic effect against tumor cells after the release of reactive oxygen species and nitric oxide. TAM M1 are characterized by a relevant expression of major histocompatibility complex proteins class II and production of interleukin 12 (IL-12) and interleukin 23 (IL-23) to induce Th1 lymphocytes response [9]. Contrary, M2 TAM are activated with transforming growth factor beta (TGF-β), interleukin 4(IL-4) and interleukin 13 (IL-13) to release a set of growth factors: VEGF, epidermal growth factor (EGF), and fibroblast growth factor (FGF); thus promoting angiogenesis and tumor growth [10]. Hypoxia, colony stimulating factor 1 (CSF-1), TGF- β, IL-4 and IL-13 are able to enhance the switch of TAM from M1 to M2 phenotype, thus changing the M1/M2 ratio while tumor progression [11]. HIF-1α induces transcription of C–X–C motif receptor-4 (CXCR-4) and its specific C–X–C motif ligand 12 (CXCL12). The interaction between CXCR-4 and CXCL12 enhances the concentration of macrophages within the hypoxic area of a tumor [12]. Some authors reported also a HIF-1α-dependent transcription of VEGF-A within macrophages [13]. However, it should be stated that categorization for M1 and M2 population is a just a simplifying tool which shows the extreme poles of TAM, while TAM seem to present many intermediate phenotypes sharing both M1 and M2 markers.

One of the major stimulators for macrophage proliferation, as well as a chemotactic factor is CSF-1. Some therapeutic strategies focusing on the blockade of the CSF-1 function were used in several models of cancer, resulting in delayed cancer development together with a decreased number of TAM [9, 14]. A relevant chemotactic factor for TAM is chemokine C–C motif ligand 2 (CCL2), referred also as monocyte chemoattractant protein-1 (MCP-1), which positively correlates with TAM accumulation in many solid tumors [15, 16]. CCL2 can be released not only by TAM, but by EC, fibroblasts and tumor cells as well [17]. The most relevant transcription factor of CCL-2 is nuclear factor-κB (NFκB), which main role is associated with prevention of tumor cells, from apoptosis [18]. Tumor-derived CCL2 increases polarization of macrophages into the M2 population [19]. A blockade of CCL2 with specific antibodies in mice models showed inhibition of tumor growth, again together with the lower number of TAM [20].

TAM roles depend on their phenotype. Polarization towards M2 phenotype means that aside from the loss of the inflammatory function, M2 macrophages start to release tumorigenic and angiogenic mediators: VEGF-A, basic fibroblast growth factor (bFGF), urokinase plasminogen activator (uPA) and adrenomedullin as well as numerous proangiogenic chemokines: CXCL1, CXCL8, CXCL12, CXCL13, CCL2 and CCL5 [21, 22]. TAMs synthesize VEGF after being induced by hypoxia the in HIF-1α-related pathway, which results in vessels sprouting to avascular regions of the tumor [13, 21]. Hypoxia stimulates TAMs to synthesis of proteolytic enzymes such as matrix metalloproteases (MMP): MMP-1, MMP-7 and MMP-9 which are involved in the degradation of ECM for cell migration and release of sequestrated VEGF from ECM [23, 24]. Next, secretion of phospholipase A_2_ on site of cancer-related-inflammation results in further release of VEGF family from TAM [25].

Accumulation of TAMs in a vicinity of a tumor is a characteristic finding in gynecologic tumors as well. Prevalence of M1 or M2 TAMs seems to be correlated with the tumor aggressiveness and prognosis. In ovarian cancer tissue, high ratio of M1–M2 phenotype is associated with better prognosis. Moreover, the ratio is lowered as the disease progresses [26]. A similar relationship is found in cervical cancer, where angiogenesis is positively correlated with TAMs accumulation [27]. The number of M2 macrophages is increased due to chemokines released from tumor cells: Il-6, IL-4, VEGF, TGF-β. As these cytokines share proangiogenic properties, the neovascularization is augmented both with a direct cytokine effect and the M2 activity [28]. It seems, that lymphangiogenesis can also be stimulated by the interaction between M2 macrophages and cervical cancer cells [29]. For patients with locally advanced cervical cancer treated with chemoradiation, the low ratio of M1/M2 macrophages is a negative prognostic factor as it is correlated with poor pathological response and worse 5-year overall survival [30]. For endometrial cancer, together with a lower differentiation, indicated by low progesterone receptor expression, increased concentration of TAMs within tumor stroma is characteristic [31]. A direct association of TAMs and tumor angiogenesis was shown in the endometrial cancer: high vascularity and deeper invasion was observed in those tumors which possessed higher number of TAMs, finally resulting in lower progression-free survival [32]. Inducing repolarization back to M1 phenotype through natural products like Neferine (an alkaloid from Nelumbo nucifera Gaertn.) or deoxyschizandrin (extracted from Schisandra berries) decreased the formation of new vessels in ovarian cancer models [33, 34]. Chemokines responsible for TAMs recruitment are also active in gynecological cancers. A high concentration of CSF-1 in endometrial cancer is a prognostic factor for poor overall survival [35]. Inhibition of CSF-1 function by blockade of their specific receptor, decreases the production of ascitic fluid and concentration of M2 macrophages in mice model of ovarian cancer [36]. Another chemokine, CCL-2 was proven to be responsible for migration and adhesion of ovarian cancer cells [37]. A CCL2 level is positively correlated with TAMs concentration in ovarian cancer tissues [38]. A CCL2 transcription in ovarian cancer cells is regulated by the NFκB, which is highly increased in high-grade ovarian cancers. An inhibition of NFκB signaling suppresses angiogenesis and increases efficacy of anti-cancer therapy [39–41]. NFκB signaling is also directly associated with TAMs; specific disruption of this pathway in ovarian cancer model leads toward inversion of TAMs phenotype back to the M1 population together with tumor regression [42]. Finally, the angiogenic activity of TAMs after stimulation with phospholipase A_2_, is related to a poor prognosis of patients with ovarian cancer [43].

### Neutrophils in tumor surroundings

Neutrophils are less numerous in the tumor microenvironment than TAM, nevertheless they can influence the activity of macrophages by releasing many cytokines, thus influencing tumor growth. Aside from the “classical” role, they are proposed to be important players in malignant transformation and progression of a tumor, influencing neovascularization as well [44]. It is suggested that, while the tumor grows and acquires a more aggressive phenotype, the neutrophils in the tumor vicinity lose their cytotoxic properties and become more tumorigenic. This change is observed together with spatial relocation of neutrophils from the tumor periphery to the inner part [45]. It has been proposed to distinguish two different populations of tumor-associated neutrophils (TAN), similarly to TAM: N1 and N2 phenotype according to their anti- and pro-tumorigenic properties, anyhow some differences should be noticed. A tumor microenvironment influences TAN to acquire N2 phenotype and it is questionable if this polarization is reversible like in the TAM population [46]. TAN have a double origin which covers converted neutrophils transferred directly from bone marrow and granulocytic myeloid-derived suppressor cells (G-MDSC) attracted to the tumor, analogously to the TAM population [47]. Nevertheless, it is not clear which group is the main source for further transformation towards the N2 phenotype, mainly driven by TGF- β [48].

The recruitment of TAN is achieved through the release of specific chemokines from the tumor microenvironment—CXCL1, CXCL2; cytokines—INF- γ, TNF-α; growth factors—granulocyte colony stimulating factor (G-CSF) and presence of specific adhesion molecules on the EC surface [47, 49]. Most chemoattractants act through receptors CXCR1 an CXCR2, which are widely expressed on the neutrophils membrane. These are not only the tumor cells which participate in TAN recruitment, but others like TAM and T-regulatory lymphocytes, the latter acting through CXCL8 [50].

Neutrophils can be involved in angiogenesis by the release of largely stored intracellular VEGF following after stimulation with various factors [51]. The synthesis of VEGF mRNA is low in mature neutrophils, while it has been observed that TANs are potent to increase VEGF mRNA synthesis in the tumor microenvironment. Nevertheless, it seems that the main angiogenetic activity of neutrophils is associated with an indirect influence on the concentration of VEGF and other proangiogenic factors through extracellular matrix [52]. The release of matrix metalloproteinase-9 (MMP-9) from TANs is essential to the remodelling of ECM to facilitate the sprouting of EC. What is also important is that, MMP-9 is also involved in the deliberation of TGF-β, bFGF and VEGF, as they have been sequestrated within the ECM [53]. Blockade of Gr1^+^ positive cells with specific antibodies results in decreased neoangiogenesis in animal models [54]. Accumulation of CD11^+^Gr1^+^ neutrophils within tumor tissue is a characteristic feature of tumors, which start their resistance to anti-VEGF therapy. G-CSF released from tumor cells stimulates migration of CD11^+^Gr1^+^ neutrophils towards tumor tissue. G-CSF stimulates the release of proangiogenic factors other than VEGF, thus creating the escape from anti-VEGF therapy [55]. Bv8 or prokineticin has recently been recognized as a new proangiogenic factor. Interestingly, upregulation of Bv8 synthesis was found within TANs after stimulation with G-CSF [56]. Bv8 increases proliferation, migration and survival of EC as well as acting as a chemoattractant for neutrophils [57]. Another cytokine released by neutrophils is CXCL8 which, away from its chemotactic activity, induces proliferation and migration of EC. The main angiogenetic effect is achieved by the binding of CXCL8 to CXCR1 and CXCR2 receptors [58]. Neutrophils secrete CXCL8 after stimulation with different cytokines released from the tumor [59]. The direct interaction between TANs and neoplasm cells results in an autocrine bidirectional stimulation. Tumor cells release GM-CSF, which stimulates synthesis and release of oncostatin M by neutrophils. Oncostatin M gives the return signal to tumor cells for additional secretion of VEGF-A [60].

In gynecological tumors recruitment of TAN to tumor microenvironment is stimulated by tumor cells in a similar manner, as it is characteristic for other tumors. The presence of TANs, disregarding the angiogenetic effect, can be an independent prognostic factor in cervical carcinoma—high index of CD66b^+^ neutrophils to CD8^+^ lymphocytes was related with shorter recurrence-free survival [61]. Ovarian cancer cells provoke recruitment of TANs to the tumor microenvironment by secreting large amounts of CXCL8 (IL-8), which can bind to both CXCR1 and CXCR2 [62]. CXCL8 levels in ascites due to ovarian cancer, positively correspond to angiogenetic effectiveness [63]. Endometrial cancer cells are also positive for CXCR1 and CXCR2, and CXCL8 exerts its mitogenic effect through these receptors [64]. One of the reasons for failure in advanced ovarian cancer is the escape from antiangiogenic therapy, and both CXCL8 and Bv8 synthesis by TANs has been suggested to be one of the participants [65]. Despite the fact, that Bv8 is involved in placental development as well as in ovarian angiogenesis, there is little evidence for its participation in both endometrial and ovarian cancer, while there are some other adenocarcinomas recognized where Bv8 has been proven to exert an angiogenetic effect [66]. Bv8 is produced by stromal cells within ovarian tumors but no significant correlation with progression of disease has been found [67]. Concluding the role of TANs in tumors development, it is mainly associated with influencing the tumor microenvironment rather than individual cells. Nevertheless, it is very difficult to precisely separate the activity of TANs from other tumor-associated cells e.g. TAMs or mast cells and the role of chemokines released from TANs still remains to be well defined.

### Mast cells and basophils

Tissue mast cells together with circulating basophils possess the unique tetrameric receptor for IgE having a well-defined role in pathogenesis of allergic diseases. They produce three types of mediators: (1) pre-synthesized and stored substances like histamine, tryptase, chymase; (2) newly synthesized lipid-derived substances like leukotrienes and prostaglandins; (3) cytokines: IL-1α, IL-1β, IL-3, IL-4, IL-5, IL-6, IL-9, IL- 10, IL-12, IL-13, IL-15, IL-16, IL-18, IFN-α, TGF-β,VEGF, chemokines, nitric oxide and oxide radicals [68]. Activation of the IgE receptor or adenosine receptor, hypoxia and corticotrophin-releasing hormone (CRH) enhance the release of VEGF and IL-8. Moreover, VEGF-R1 and VEGF-R2 receptors are present on the surface of mast cells, actively participating in the chemotaxis of mast cells to the inflammatory site [69]. Peritumoral density of mast cells is increased in a similar mechanism. Chemotactic cytokines released from a tumor microenvironment i.e. CCL2, CXCL1, CXCL10, PGE_2_, histamine, VEGF, angiopoietin 1 (Ang1), CXCL8/IL-8 attract mast cells from the vicinity and recruit them from circulation [70]. However, it still remains unclear what is the exact role of mast cells in tumor development. Mast cell presence is observed especially where new vessels form, indicating their stimulatory role in neoangiogenesis [71, 72]. Mast cells can produce MMP-9 and other proteolytic enzymes, specifically chymase and tryptase, which are able to digest ECM to facilitate tumor invasion [73]. Activation of mast cells coincides with the angiogenic switch in squamous carcinomas [74]. Activation of mast cells is done by tumor cells, other immunocompetent cells in the vicinity and, finally, on the autocrine way by themselves. First hypoxia, a distinctive feature of tumor, stimulates a release of VEGF and IL-6 from mast cells [75]. Adenosine released from tumor cells and mast cells, and PGE_2_ induced by tumor cyclooxygenase increase histamine synthesis as well as VEGF and other angiogenic cytokines in mast cells [76].

Both human and experimental models show different results in different types of cancer. Mast cell density was described as an indicator of poor prognosis in endometrial and cervical cancer, while one study concerning ovarian carcinoma showed that increased mast cell density was associated with better prognosis [77, 78]. Concerning the fact that mast cells release VEGF and other proangiogenic cytokines, it comes as no surprise that their presence supports a development of a tumor. The mean vessels density positively correlates with mast cells density and tumor progression in both the cervical dysplasia and carcinoma [79]. A less differentiated type of endometrial cancer, when compared to well-differentiated types, is characterized with increased density of mast cells and a closer location of mast cells to the blood vessel. Some experimental studies confirm the stimulatory effect of the mast cell histamine on tumor cells invasive ability: HPV18-positive cervical carcinoma cells migration was accelerated in the presence of mast cells and inhibited with histamine receptor − 1 (H_1_R) inhibitors [80]. On the other hand, some authors did not prove the clinical correlation between the number of mast cells, stage of the disease and the development of a vessel network in endometrial cancer [81]. It should be stated, however, that the study was done on a relatively small number of cases which could decrease the statistic power of the study. The role of mast cells in tumor development, however, needs further investigation. It seems to be mostly associated with the provision of pro-angiogenic factors which facilitate tumor growth.

### Tumor-infiltrating lymphocytes

The emerging role of lymphocytes infiltrating the tumor tissue (TILs—tumor-infiltrating lymphocytes) provides modern oncology with an increasing number of proofs for anti-tumor T-cell response, better than other non-infiltrating lymphocytes. TILs are mainly represented by CD3+ CD4+ (helper) and CD3+ CD8+ (cytotoxic) T cells [82]. Generally, in most of solid tumors, TILs are responsible for direct cytotoxic activity and indirect activation of immunologic response against tumor cells. Their presence is a positive predictor of response to chemotherapy and good prognostic factor for overall survival (OS) in many tumors like: neck and head squamous carcinoma, melanoma, lung cancer, breast cancer [83, 84].

Li et al. published a meta-analysis of 21 studies including the overall number of 2903 patients with ovarian cancer and found a significant correlation between some types of TILs and both progression-free survival (PFS) and overall survival (OS). Specifically, the presence of CD3+ and CD8+ TILs in epithelial tissue was a good prognostic marker, while the stromal TILs had no impact on PFS nor OS [85]. These “good” lymphocytes trigger an immunologic response against tumor cells mainly by direct cell-to-cell contact, being supported by another subtype of lymphocytes—CD103+ [86]. The activity of TILs in cervical cancer and endometrial cancer is less known. However, it is postulated, that decreased survival in cervical cancer is associated with the domination of Treg FoxP3+ lymphocytes over CD103+ lymphocytes, while the latter are a good biomarker for HPV-targeted immunotherapy [87, 88]. A similar finding was found for endometrial cancer. An infiltration of tumor tissue with FoxP3+ lymphocytes positively correlated with vessel density although there was no significant impact on prognosis [89]. The presence of CD103+ lymphocytes in endometrial tumors were associated with improved prognosis and this effect was especially characteristic for high-risk endometrial cancer [89]. However, most of the authors highlight the direct activity of TILs against cancer cells, not their antiangiogenic effect. The association between proangiogenic activity of some subpopulations of TILs, like FoxP3+, is rather due to the co-occurrence of the immunologic switch together with the progression of a tumor than its direct influence of TILs to angiogenesis.

## The role of extracellular matrix

Components of ECM serve as a scaffold for many cells (tumor, macrophages and endothelial) to create a structural support for them. Nevertheless, the role of ECM components is much more complex as individual glycoproteins of ECM are actively involved in cell-to-cell interactions including the effect of immune cells on angiogenesis. The summation of the role of ECM proteins on the angiogenesis is given in Table 2. Proteolytic fragments of elastin created by MMP-9, denatured collagen I and fibronectin are chemoattractants for other monocytes and macrophages [90, 91].

**Table 2:** The compounds of extracellular matrix that participate in angiogenesis and their role in gynecological tumors

ECM protein	Cellular receptor	Angiogenic effect	Tumor type and specific role	References
Tenascin SIBLING CCN family	αvβ3-integrin in EC	Stimulation of endothelial cells proliferation and survival	Ovarian cancer—worse prognosis Cervical cancer—increased angiogenesis Endometrial cancer—worse prognosis	[97] [99, 102] [104] [103]
Osteopontin	α9β1-integrin in TAM	Inducing of cyclooxygenase expression		[98]
	α9β1-integrin in ovarian cancer cells	Activation of the PI3-K/Akt pathway	Ovarian cancer—promotes tumor survival Cervical cancer—increases invasiveness	[100] [101]
LMWHA	TLR-2 and TLR-4 in macrophages	Increasing the synthesis of MMP-12, IL-10 and IL-12		[107]
	CD44 and receptor for HA-mediated motility in endothelial cells	Stimulation of EC proliferation and their increased motility		[108]
			Mucinous and clear cell ovarian cancer—high activity of hyaluronidase Endometrial and serous ovarian cancer, endometrioid endometrial cancer—low activity of hyaluronidase	[113] [114] [115]
Fibronectin	α5β1-integrin αvβ3-integrin both in EC	Prolongation of EC survival	Ovarian cancer—worse prognosis	[118] [121]
Thrombospondin	CD36 and CD47 both in EC	Negative: inhibits the proliferation of EC and stimulate their apoptosis	Negative correlation with tumor aggressiveness	[124] [125] [129]
	α6β1-integrin in TAM	Negative: polarization towards M1 type		[130]

### Collagen

Formerly, collagen was thought to be a natural barrier against tumor invasion. However, these tumors which are characterized with increased collagen synthesis are more aggressive and rich in blood vessels, thus suggesting that collagen rather promotes invasiveness and angiogenesis [92]. It is observed that, during growth of ovarian cancer implants, TAMs upregulate genes that are responsible for ECM remodeling, namely lumican and lysyl oxidase [93]. They are both involved in the organization of collagen structure and cross-linking of ECM proteins [94].

Reorganization of collagen structure has been proved to be associated with poor prognosis of serous ovarian cancer patients [95]. An interesting finding was provided by Ames at al. They found that RDG collagen epitope, generated during proteolytic degradation of ECM, may be responsible for the induction of angiogenesis and inflammation. This can be achieved by the stimulation of mechanical activation of αvβ3 integrin, next the Src-dependent phosphorylation of p38 MAPK (mitogen-activated protein kinase) and finally, the promotion of nuclear accumulation of the Yes-associated protein (YAP) thus enhancing endothelial cell growth [96].

### Integrin ligands

Integrins are common transmembrane receptors consisting of α- and β-subunits that mediate cell-to-cell and cell-to-ECM adhesive interactions. Nevertheless, their role is not limited to a structural function. They also mediate transduction of signals from the ECM to the cell interior and backwards. They participate in angiogenesis, lymphangiogenesis, migration, growth and survival [97]. ECM provide the tumor microenvironment with a rich source of proteins that bind with integrins responsible for angiogenesis. These are: tenascins, small integrin-binding ligand N-linked glycoproteins (SIBLING) and CCN [which is the acronym for cysteine-rich protein 61 (CYR61), connective tissue growth factor (CTGF) and the nephroblastoma overexpressed (NOV)] family. They mostly play with αvβ3-integrins and mediate proliferation and survival of EC acting together with the VEGF-1 and VEGF-2 signalling pathways [97]. Osteopontin, one of the SIBLINGs family stimulates angiogenesis directly by upregulation of cyclooxygenase expression in TAMs [98].

A high tenascin expression was shown in many cancers including ovarian and correlated with a poor prognosis [99]. Besides proangiogenic activity, osteopontin promotes survival of ovarian cancer cells by the activation of the PI3-K/Akt pathway [100] and is associated with invasiveness of cervical cancer [101]. CCN1 mRNA and protein levels are increased in ovarian cancer cells as well as in endometrial carcinoma and indicative of a poor prognosis [102, 103]. Expression of CCN3 assessed in cervical cancer cells was positively correlated with vascularization, stage of the disease and lymph node involvement [104].

### Hyaluronic acid

A component of ECM, hyaluronic acid (HA), participates in differentiation of TAM towards the M2 phenotype [105]. In vitro research, it was discovered that tumors rich in fibroblasts deprived of HA synthesis showed decreased infiltration with monocytes and TAM [106]. It should be clearly stated that pro-inflammatory properties of HA are specific for the subtype of HA which is a low molecular weight hyaluronic acid (LMWHA)—a product of HA degradation. LMWHA increases the synthesis of MMP-12, IL-10 and IL-12 by macrophages acting mainly through toll-like receptor (TLR)-2 and TLR-4 [107]. The direct role of LMWHA in angiogenesis is associated with stimulation of EC proliferation and their increased motility. Two main receptors for HA are involved in these processes: CD44 (variant 8–10) and RHAMM (Receptor for HA-mediated motility) [108]. Contrary to LMWHA, high molecular weight HA (HMWHA) seems to play an anti-angiogenic role by maintaining vascular barrier integrity [109]. HMWHA acts through CD44 receptors but not through 8–10 variants [110]. Overexpression of HA synthase, resulting in overproduction of HMWHA, is associated with lower tumor growth but only in cases where the activity of hyaluronidase is low. Over co-expression of both HA synthase and hyaluronidase activates the process of tumor angiogenesis, thus supporting the opposite function of HMWHA and LMWHA [111]. Macrophages can also stimulate the degradation of HMWHA through a combined action of hyaluronidase and reactive oxygen species [112]. However, the ratio of degradation can differ between histologic types of cancer.

In ovarian cancer, mucinous and clear cell carcinoma are characterized with a high activity of hyaluronidase, while in ovarian high-grade serous carcinoma the activity of hyaluronidase is low [113, 114]. A similar observation was made in endometrial endometrioid cancer, where the level of HA synthase was stable and hyaluronidase activity was reduced, thus resulting in a high concentration of HA [115]. Overall, the level of HA in the tumor-associated angiogenesis could be an important factor but larger studies are necessary to understand its significance.

### Fibronectin

Fibronectin, another compound of ECM, is typically involved in fetal vasculogenesis and rarely found in an adult vascular network. However, under pathologic conditions, fibronectin synthesis starts again [116]. Fibronectin binds to the α_5_β_1_ integrin which is upregulated during tumor angiogenesis [117]. Activation of both α_5_β_1_ and α_v_β_3_ integrin with fibronectin leads to prolongation of EC survival and is necessary in vascular morphogenesis [118]. Interestingly, the migration of EC is achieved after activation of FGF receptor-1 by fibronectin together with β_1_ integrin [119].

In metastatic ovarian cancer, fibronectin is released from mesothelial cells after their stimulation with TGF-β as only mesothelial cells undergo a process of endothelial to mesenchymal transition (EMT) [120]. Fibronectin is elevated in advanced ovarian cancer and its level is associated with a poor prognosis [121]. The EMT process can be activated by the ovarian cancer cells alone and by another inflammatory cytokines from myeloid-derived cells [120, 122].

### Thrombospondin

Thrombospondins stay on the other side of angiogenesis, being a potent inhibitor of this process. They are ECM glycoproteins, produced by fibroblasts, endothelial cells, blood platelets and immune cells [123]. Thrombospondins act mainly through CD36 and CD47 to inhibit the proliferation of EC and stimulate their apoptosis [124, 125]. Thrombospondin-1 is a positive modulator of antitumor immune activity by increasing the M1 number in the TAM population [126]. Contrary, an intensive inflammatory process deactivates thrombospondin-1 by secretion of neutrophils proteases. This is especially interesting for metastatic growth in different organs. Ablation of neutrophil proteases by genetic modification decreased the number of lung metastases [127].

The level of thrombospondin-1 negatively correlates with the level of vascularization and aggressiveness in endometrial cancer [128]. Similar findings are characteristic of ovarian carcinoma cells. The aggressiveness is higher with a decreasing level of thrombospondins expression [129, 130]. It seems that such an effect is not only associated with intensive angiogenesis in the absence of thrombospondins but with the switch from M1 to M2 TAM population as well.

Extracellular matrix is, therefore, not only the scaffold for creation of new vessels and cellular migration, but its components are actively embedded in complex processes between inflammation, new vessels creation and tumor growth.

## Summary

The process of angiogenesis is complex, and therefore, it is extremely difficult to distinguish a role of any single factor.

The most dominant immunocompetent cells are macrophages with two opposite phenotypes of which, M2 are associated with secretion of proangiogenic cytokines, mainly VEGF and others like Il-6, IL-4, TGF-β. The dual role of these cytokines associate proangiogenic with their chemotactic properties, thus augmenting the inflammation process inside the tumor parallel to angiogenesis. Generally, for gynecological tumors, there is a positive correlation between the concentration of macrophages, angiogenesis and advancement of a disease. Blockade of transcription factors like NFκB, which are overstimulated with angiogenic cytokines, leads to a reversion of the inflammatory process towards “anti-tumor” behavior, as it was proven in ovarian cancer.

The correspondent behavior is characteristic for neutrophils. Similarly, two distinct populations can be distinguished and N2 represents the protumorigenic one. The angiogenic feature of N2 neutrophils is associated not only with a direct secretion of cytokines like VEGF, CXCL8, but also deliberation of these cytokines from ECM after digestion the ECM with MMP-9. In ovarian cancer, as well as in endometrial cancer, dualistic angiogenic and mitogenic role of TANs cytokines is observed. The diversity of different cytokines released by TANs is the possible explanation for failure in antiangiogenic treatment based on anti-VEGF therapy.

Mast cells, which are widely known as proangiogenic cells, release many proangiogenic cytokines as well as MMP-9, releasing VEGF from ECM in the same way as neutrophils. Despite some controversies, it seems that mast cells presence is positively correlating with tumor growth especially in cervical cancer. In experimental studies, blockade of H_1_R decreased invasiveness of HPV-related cervical carcinoma.

TILs role depends on their phenotype, with CD103^+^ being a good prognostic factor in endometrial and cervical cancer. Nevertheless, their contribution in tumor inhibition is rather associated with direct activity than antiangiogenic properties.

The role of another important player should be raised, which is the ECM. Components of ECM are not only the passive scaffold for the cells, but may play an active role as chemokines and angiogenesis stimulators.

Fragments of collagen, formed after TAMs and N2 activity augment growth of endothelial cells and their concentration is related with poor prognosis of ovarian cancer patients. Next, increased concentration of integrin activators like tenascins, SIBLINGs and CCN leads to increased angiogenesis, especially in ovarian and advanced endometrial cancer.

LMWHA increases infiltration of tumor tissue by M2 macrophages, as well as stimulates endothelial cells proliferation and migration. Activation of hyaluronidase by macrophages, and sequential increase of LMWHA is observed in specific ovarian cancer subtypes like mucinous and clear cell carcinoma, contrary to serous carcinoma. The role of hyaluronidase in ovarian cancer development still need clarification.

Fibronectin, usually active in fetal development only, is reactivated after stimulation of mesothelial cells with TGF-β released from active macrophages. Fibronectin stimulates both α_5_β_1_ and α_v_β_3_ integrin in endothelial cells, thus increasing angiogenesis. The elevated level of fibronectin is associated with progression of ovarian cancer.

The inhibitor of angiogenesis—thrombospondin, is deactivated after activation of neutrophils. The level of thrombospondin is negatively correlated with aggressiveness of both endometrial and ovarian cancer.

Summing up, inflammatory process seems to be an inseparable part of a tumor’s growth—both as a response of the host against the tumor and stimulated by the tumor itself. Cytokines, growth factors, chemokines etc. released by immunocompetent cells can provide a side-effect of stimulating angiogenesis and further tumor progression. Inhibition of the inflammatory process, or reversing back the cells towards anti-tumor phenotype, could give another advantage with inhibiting the angiogenesis. The development of gynecological cancers, as it has been shown, is associated with increased vascularization, thus the pathogenesis of vascular network development is especially interesting for new concepts of anti-tumor therapies, if possibly targeted, without a toxic effect to other systems.

## Abbreviations

bFGF: Basic fibroblast growth factor
CCL2: Chemokine (C–C motif) ligand 2
CCN: CYR61, CTGF, NOV
CRH: Corticotrophin-releasing hormone
CSF-1: Colony stimulating factor 1
CTGF: Connective tissue growth factor
CXCL12: C–X–C motif ligand 12
CXCR-4: C–X–C motif receptor-4
CYR61: Cysteine-rich protein 61
ECM: Extracellular matrix
EMT: Endothelial to mesenchymal transition
G-CSF: Granulocyte colony stimulating factor
H_1_R: Histamine receptor-1
HA: Hyaluronic acid
HIF-1α: Hypoxia-inducible factor-1α
HMWHA: High molecular weight hyaluronic acid
IL: Interleukin
INF-γ: Interferon-γ
LMWHA: Low molecular weight hyaluronic acid
MAPK: Mitogen-activated protein kinase
MCP-1: Monocyte chemoattractant protein-1
MMP: Matrix metalloproteases
NFκB: Nuclear factor-κB
NOV: Nephroblastoma overexpressed
OS: Overall survival
PFS: Progression-free survival
RHAMM: Receptor for hyaluronic acid-mediated motility
SIBLING: Small integrin-binding ligand N-linked glycoproteins
TAM: Tumor-associated macrophages
TAN: Tumor-associated neutrophils
TGF-β: Transforming growth factor beta
TIE2: Tyrosine kinase with immunoglobulin-like and EGF-like domains 2
TILs: Tumor-infiltrating lymphocytes
TNF-α: Tumor necrosis factor-α
uPA: Urokinase plasminogen activator
VEGF: Vascular endothelial growth factor
VEGFR-1: Vascular endothelial growth factor receptor-1
YAP: Yes-associated protein

## References

[1] Folkman J (1990). What is the evidence that tumors are angiogenesis dependent?. J Natl Cancer Inst.

[2] Hickey MM, Simon MC (2006). Regulation of angiogenesis by hypoxia and hypoxia-inducible factors. Curr Top Dev Biol.

[3] Mantovani A, Allavena P, Sica A, Balkwill F (2008). Cancer-related inflammation. Nature.

[4] Costa C, Incio J, Soares R (2007). Angiogenesis and chronic inflammation: cause or consequence?. Angiogenesis.

[5] Zhang L, Conejo-Garcia JR, Katsaros D (2003). Intratumoral T cells, recurrence, and survival in epithelial ovarian cancer. N Engl J Med.

[6] Kim J, Bae J-S. (2016) Tumor-associated macrophages and neutrophils in tumor microenvironment. Mediators Inflamm 2016:6058147. 10.1155/2016/6058147 PMC4757693 26966341

[7] Biswas SK, Mantovani A (2010). Macrophage plasticity and interaction with lymphocyte subsets: cancer as a paradigm. Nat Immunol.

[8] Movahedi K, Laoui D, Gysemans C (2010). Different tumor microenvironments contain functionally distinct subsets of macrophages derived from Ly6C(high) monocytes. Cancer Res.

[9] Qian B-Z, Pollard JW (2010). Macrophage diversity enhances tumor progression and metastasis. Cell.

[10] Lewis CE, Pollard JW (2006). Distinct role of macrophages in different tumor microenvironments. Cancer Res.

[11] Noy R, Pollard JW (2014). Tumor-associated macrophages: from mechanisms to therapy. Immunity.

[12] Schioppa T, Uranchimeg B, Saccani A (2003). Regulation of the chemokine receptor CXCR4 by hypoxia. J Exp Med.

[13] Eubank TD, Roda JM, Liu H, O’Neil T, Marsh CB (2011). Opposing roles for HIF-1α and HIF-2α in the regulation of angiogenesis by mononuclear phagocytes. Blood.

[14] Ries CH, Cannarile MA, Hoves S (2014). Targeting tumor-associated macrophages with anti-CSF-1R antibody reveals a strategy for cancer therapy. Cancer Cell.

[15] Zhang J, Lu Y, Pienta KJ (2010). Multiple roles of chemokine (C–C motif) ligand 2 in promoting prostate cancer growth. J Natl Cancer Inst.

[16] Qian B-Z, Li J, Zhang H, Kitamura T, Zhang J, Campion LR, Kaiser EA, Snyder LA, Pollard JW (2011). CCL2 recruits inflammatory monocytes to facilitate breast-tumour metastasis. Nature.

[17] Ono M (2008). Molecular links between tumor angiogenesis and inflammation: inflammatory stimuli of macrophages and cancer cells as targets for therapeutic strategy. Cancer Sci.

[18] Escárcega RO, Fuentes-Alexandro S, García-Carrasco M, Gatica A, Zamora A (2007). The transcription factor nuclear factor-kappa B and cancer. Clin Oncol (R Coll Radiol).

[19] Sierra-Filardi E, Nieto C, Domínguez-Soto A (2014). CCL2 shapes macrophage polarization by GM-CSF and M-CSF: identification of CCL2/CCR2-dependent gene expression profile. J Immunol.

[20] Mizutani K, Sud S, McGregor NA, Martinovski G, Rice BT, Craig MJ, Varsos ZS, Roca H, Pienta KJ (2009). The chemokine CCL2 increases prostate tumor growth and bone metastasis through macrophage and osteoclast recruitment. Neoplasia.

[21] Chanmee T, Ontong P, Konno K, Itano N (2014). Tumor-associated macrophages as major players in the tumor microenvironment. Cancers (Basel).

[22] Goswami KK, Ghosh T, Ghosh S, Sarkar M, Bose A, Baral R (2017). Tumor promoting role of anti-tumor macrophages in tumor microenvironment. Cell Immunol.

[23] Burke B, Giannoudis A, Corke KP, Gill D, Wells M, Ziegler-Heitbrock L, Lewis CE (2003). Hypoxia-induced gene expression in human macrophages: implications for ischemic tissues and hypoxia-regulated gene therapy. Am J Pathol.

[24] Murdoch C, Lewis CE (2005). Macrophage migration and gene expression in response to tumor hypoxia. Int J Cancer.

[25] Granata F, Frattini A, Loffredo S (2010). Production of vascular endothelial growth factors from human lung macrophages induced by group IIA and group X secreted phospholipases A2. J Immunol.

[26] Zhang M, He Y, Sun X, Li Q, Wang W, Zhao A, Di W (2014). A high M1/M2 ratio of tumor-associated macrophages is associated with extended survival in ovarian cancer patients. J Ovarian Res.

[27] Jiang S, Yang Y, Fang M, Li X, Yuan X, Yuan J (2016). Co-evolution of tumor-associated macrophages and tumor neo-vessels during cervical cancer invasion. Oncol Lett.

[28] Pedraza-Brindis EJ, Sánchez-Reyes K, Hernández-Flores G, Bravo-Cuellar A, Jave-Suárez LF, Aguilar-Lemarroy A, Gómez-Lomelí P, López-López BA, Ortiz-Lazareno PC (2016). Culture supernatants of cervical cancer cells induce an M2 phenotypic profile in THP-1 macrophages. Cell Immunol.

[29] Ding H, Cai J, Mao M (2014). Tumor-associated macrophages induce lymphangiogenesis in cervical cancer via interaction with tumor cells. APMIS.

[30] Petrillo M, Zannoni GF, Martinelli E, Pedone Anchora L, Ferrandina G, Tropeano G, Fagotti A, Scambia G (2015). Polarisation of tumor-associated macrophages toward M2 phenotype correlates with poor response to chemoradiation and reduced survival in patients with locally advanced cervical cancer. PLoS ONE.

[31] Jiang X, Tang Q, Li H, Shen X, Luo X, Wang X, Lin Z (2013). Tumor-associated macrophages correlate with progesterone receptor loss in endometrial endometrioid adenocarcinoma. J Obstet Gynaecol Res.

[32] Soeda S, Nakamura N, Ozeki T, Nishiyama H, Hojo H, Yamada H, Abe M, Sato A (2008). Tumor-associated macrophages correlate with vascular space invasion and myometrial invasion in endometrial carcinoma. Gynecol Oncol.

[33] Zhang Q, Li Y, Miao C (2018). Anti-angiogenesis effect of Neferine via regulating autophagy and polarization of tumor-associated macrophages in high-grade serous ovarian carcinoma. Cancer Lett.

[34] Lee K, Ahn J-H, Lee K-T, Jang DS, Choi J-H (2018). Deoxyschizandrin, isolated from Schisandra berries, induces cell cycle arrest in ovarian cancer cells and inhibits the protumoural activation of tumour-associated macrophages. Nutrients.

[35] Smith HO, Stephens ND, Qualls CR, Fligelman T, Wang T, Lin C-Y, Burton E, Griffith JK, Pollard JW (2013). The clinical significance of inflammatory cytokines in primary cell culture in endometrial carcinoma. Mol Oncol.

[36] Moughon DL, He H, Schokrpur S, Jiang ZK, Yaqoob M, David J, Lin C, Iruela-Arispe ML, Dorigo O, Wu L (2015). Macrophage blockade using CSF1R inhibitors reverses the vascular leakage underlying malignant ascites in late-stage epithelial ovarian cancer. Cancer Res.

[37] Furukawa S, Soeda S, Kiko Y (2013). MCP-1 promotes invasion and adhesion of human ovarian cancer cells. Anticancer Res.

[38] Negus RP, Stamp GW, Relf MG, Burke F, Malik ST, Bernasconi S, Allavena P, Sozzani S, Mantovani A, Balkwill FR (1995). The detection and localization of monocyte chemoattractant protein-1 (MCP-1) in human ovarian cancer. J Clin Invest.

[39] Huang S, Robinson JB, Deguzman A, Bucana CD, Fidler IJ (2000). Blockade of nuclear factor-kappaB signaling inhibits angiogenesis and tumorigenicity of human ovarian cancer cells by suppressing expression of vascular endothelial growth factor and interleukin 8. Cancer Res.

[40] Leizer AL, Alvero AB, Fu HH, Holmberg JC, Cheng Y-C, Silasi D-A, Rutherford T, Mor G (2011). Regulation of inflammation by the NF-κB pathway in ovarian cancer stem cells. Am J Reprod Immunol.

[41] Mabuchi S, Ohmichi M, Nishio Y (2004). Inhibition of NFkappaB increases the efficacy of cisplatin in in vitro and in vivo ovarian cancer models. J Biol Chem.

[42] Hagemann T, Lawrence T, McNeish I, Charles KA, Kulbe H, Thompson RG, Robinson SC, Balkwill FR (2008). “Re-educating” tumor-associated macrophages by targeting NF-kappaB. J Exp Med.

[43] Gorovetz M, Baekelandt M, Berner A, Trope’ CG, Davidson B, Reich R (2006). The clinical role of phospholipase A2 isoforms in advanced-stage ovarian carcinoma. Gynecol Oncol.

[44] Mantovani A, Cassatella MA, Costantini C, Jaillon S (2011). Neutrophils in the activation and regulation of innate and adaptive immunity. Nat Rev Immunol.

[45] Mishalian I, Bayuh R, Levy L, Zolotarov L, Michaeli J, Fridlender ZG (2013). Tumor-associated neutrophils (TAN) develop pro-tumorigenic properties during tumor progression. Cancer Immunol Immunother.

[46] Piccard H, Muschel RJ, Opdenakker G (2012). On the dual roles and polarized phenotypes of neutrophils in tumor development and progression. Crit Rev Oncol Hematol.

[47] Fridlender ZG, Sun J, Mishalian I (2012). Transcriptomic analysis comparing tumor-associated neutrophils with granulocytic myeloid-derived suppressor cells and normal neutrophils. PLoS ONE.

[48] Fridlender ZG, Sun J, Kim S, Kapoor V, Cheng G, Ling L, Worthen GS, Albelda SM (2009). Polarization of tumor-associated neutrophil phenotype by TGF-beta: “N1” versus “N2” TAN. Cancer Cell.

[49] Fridlender ZG, Albelda SM (2012). Tumor-associated neutrophils: friend or foe?. Carcinogenesis.

[50] Himmel ME, Crome SQ, Ivison S, Piccirillo C, Steiner TS, Levings MK (2011). Human CD4 + FOXP3 + regulatory T cells produce CXCL8 and recruit neutrophils. Eur J Immunol.

[51] Gaudry M, Brégerie O, Andrieu V, El Benna J, Pocidalo MA, Hakim J (1997). Intracellular pool of vascular endothelial growth factor in human neutrophils. Blood.

[52] Tazzyman S, Niaz H, Murdoch C (2013). Neutrophil-mediated tumour angiogenesis: subversion of immune responses to promote tumour growth. Semin Cancer Biol.

[53] Shuman Moss LA, Jensen-Taubman S, Stetler-Stevenson WG (2012). Matrix metalloproteinases: changing roles in tumor progression and metastasis. Am J Pathol.

[54] Nozawa H, Chiu C, Hanahan D (2006). Infiltrating neutrophils mediate the initial angiogenic switch in a mouse model of multistage carcinogenesis. Proc Natl Acad Sci USA.

[55] Qu X, Zhuang G, Yu L, Meng G, Ferrara N (2012). Induction of Bv8 expression by granulocyte colony-stimulating factor in CD11b + Gr1 + cells: key role of Stat3 signaling. J Biol Chem.

[56] Zhong C, Qu X, Tan M, Meng YG, Ferrara N (2009). Characterization and regulation of bv8 in human blood cells. Clin Cancer Res.

[57] Shojaei F, Wu X, Zhong C (2007). Bv8 regulates myeloid-cell-dependent tumour angiogenesis. Nature.

[58] Murdoch C, Monk PN, Finn A (1999). Cxc chemokine receptor expression on human endothelial cells. Cytokine.

[59] Scapini P, Lapinet-Vera JA, Gasperini S, Calzetti F, Bazzoni F, Cassatella MA (2000). The neutrophil as a cellular source of chemokines. Immunol Rev.

[60] Queen MM, Ryan RE, Holzer RG, Keller-Peck CR, Jorcyk CL (2005). Breast cancer cells stimulate neutrophils to produce oncostatin M: potential implications for tumor progression. Cancer Res.

[61] Carus A, Ladekarl M, Hager H, Nedergaard BS, Donskov F (2013). Tumour-associated CD66b + neutrophil count is an independent prognostic factor for recurrence in localised cervical cancer. Br J Cancer.

[62] Waugh DJJ, Wilson C (2008). The interleukin-8 pathway in cancer. Clin Cancer Res.

[63] Gawrychowski K, Szewczyk G, Skopińska-Różewska E, Małecki M, Barcz E, Kamiński P, Miedzińska-Maciejewska M, Śmiertka W, Szukiewicz D, Skopiński P (2014). The angiogenic activity of ascites in the course of ovarian cancer as a marker of disease progression. Dis Markers.

[64] Ewington L, Taylor A, Sriraksa R, Horimoto Y, Lam EW-F, El-Bahrawy MA (2012). The expression of interleukin-8 and interleukin-8 receptors in endometrial carcinoma. Cytokine.

[65] Hansen JM, Coleman RL, Sood AK (2016). Targeting the tumour microenvironment in ovarian cancer. Eur J Cancer.

[66] Curtis VF, Wang H, Yang P, McLendon RE, Li X, Zhou Q-Y, Wang X-F (2013). A PK2/Bv8/PROK2 antagonist suppresses tumorigenic processes by inhibiting angiogenesis in glioma and blocking myeloid cell infiltration in pancreatic cancer. PLoS ONE.

[67] Monnier J, Samson M (2010). Prokineticins in angiogenesis and cancer. Cancer Lett.

[68] Bachelet I, Levi-Schaffer F (2007). Mast cells as effector cells: a co-stimulating question. Trends Immunol.

[69] Detoraki A, Granata F, Staibano S, Rossi FW, Marone G, Genovese A (2010). Angiogenesis and lymphangiogenesis in bronchial asthma. Allergy.

[70] Varricchi G, Galdiero MR, Loffredo S, Marone G, Iannone R, Marone G, Granata F (2017). Are mast cells MASTers in cancer?. Front Immunol.

[71] Guidolin D, Marinaccio C, Tortorella C, Annese T, Ruggieri S, Finato N, Crivellato E, Ribatti D (2017). Non-random spatial relationships between mast cells and microvessels in human endometrial carcinoma. Clin Exp Med.

[72] Ribatti D, Finato N, Crivellato E, Marzullo A, Mangieri D, Nico B, Vacca A, Beltrami CA (2005). Neovascularization and mast cells with tryptase activity increase simultaneously with pathologic progression in human endometrial cancer. Am J Obstet Gynecol.

[73] Benítez-Bribiesca L, Wong A, Utrera D, Castellanos E (2001). The role of mast cell tryptase in neoangiogenesis of premalignant and malignant lesions of the uterine cervix. J Histochem Cytochem.

[74] Coussens LM, Raymond WW, Bergers G, Laig-Webster M, Behrendtsen O, Werb Z, Caughey GH, Hanahan D (1999). Inflammatory mast cells up-regulate angiogenesis during squamous epithelial carcinogenesis. Genes Dev.

[75] Gulliksson M, Carvalho RFS, Ullerås E, Nilsson G (2010). Mast cell survival and mediator secretion in response to hypoxia. PLoS ONE.

[76] Detoraki A, Staiano RI, Granata F, Giannattasio G, Prevete N, de Paulis A, Ribatti D, Genovese A, Triggiani M, Marone G (2009). Vascular endothelial growth factors synthesized by human lung mast cells exert angiogenic effects. J Allergy Clin Immunol.

[77] Faustino-Rocha AI, Ferreira R, Gama A, Oliveira PA, Ginja M (2017). Antihistamines as promising drugs in cancer therapy. Life Sci.

[78] Chan JK, Magistris A, Loizzi V, Lin F, Rutgers J, Osann K, DiSaia PJ, Samoszuk M (2005). Mast cell density, angiogenesis, blood clotting, and prognosis in women with advanced ovarian cancer. Gynecol Oncol.

[79] Utrera-Barillas D, Castro-Manrreza M, Castellanos E, Gutiérrez-Rodríguez M, Arciniega-Ruíz de Esparza O, García-Cebada J, Velazquez JR, Flores-Reséndiz D, Hernández-Hernández D, Benítez-Bribiesca L (2010). The role of macrophages and mast cells in lymphangiogenesis and angiogenesis in cervical carcinogenesis. Exp Mol Pathol.

[80] Rudolph MI, Boza Y, Yefi R, Luza S, Andrews E, Penissi A, Garrido P, Rojas IG (2008). The influence of mast cell mediators on migration of SW756 cervical carcinoma cells. J Pharmacol Sci.

[81] Pansrikaew P, Cheewakriangkrai C, Taweevisit M, Khunamornpong S, Siriaunkgul S (2010). Correlation of mast cell density, tumor angiogenesis, and clinical outcomes in patients with endometrioid endometrial cancer. Asian Pac J Cancer Prev.

[82] Badalamenti G, Fanale D, Incorvaia L (2018). Role of tumor-infiltrating lymphocytes in patients with solid tumors: Can a drop dig a stone?. Cell Immunol.

[83] de Ruiter EJ, Ooft ML, Devriese LA, Willems SM (2017). The prognostic role of tumor infiltrating T-lymphocytes in squamous cell carcinoma of the head and neck: A systematic review and meta-analysis. Oncoimmunology.

[84] Dieci MV, Mathieu MC, Guarneri V, Conte P, Delaloge S, Andre F, Goubar A (2015). Prognostic and predictive value of tumor-infiltrating lymphocytes in two phase III randomized adjuvant breast cancer trials. Ann Oncol.

[85] Li J, Wang J, Chen R, Bai Y, Lu X (2017). The prognostic value of tumor-infiltrating T lymphocytes in ovarian cancer. Oncotarget.

[86] Bösmüller H-C, Wagner P, Peper JK (2016). Combined immunoscore of CD103 and CD3 identifies long-term survivors in high-grade serous ovarian cancer. Int J Gynecol Cancer.

[87] Shang B, Liu Y, Jiang S, Liu Y (2015). Prognostic value of tumor-infiltrating FoxP3 + regulatory T cells in cancers: a systematic review and meta-analysis. Sci Rep.

[88] Komdeur FL, Prins TM, van de Wall S, Plat A, Wisman GBA, Hollema H, Daemen T, Church DN, de Bruyn M, Nijman HW (2017). CD103 + tumor-infiltrating lymphocytes are tumor-reactive intraepithelial CD8 + T cells associated with prognostic benefit and therapy response in cervical cancer. Oncoimmunology.

[89] Giatromanolaki A, Bates GJ, Koukourakis MI, Sivridis E, Gatter KC, Harris AL, Banham AH (2008). The presence of tumor-infiltrating FOXP3 + lymphocytes correlates with intratumoral angiogenesis in endometrial cancer. Gynecol Oncol.

[90] Houghton AM, Quintero PA, Perkins DL, Kobayashi DK, Kelley DG, Marconcini LA, Mecham RP, Senior RM, Shapiro SD (2006). Elastin fragments drive disease progression in a murine model of emphysema. J Clin Invest.

[91] Solinas G, Schiarea S, Liguori M (2010). Tumor-conditioned macrophages secrete migration-stimulating factor: a new marker for M2-polarization, influencing tumor cell motility. J Immunol.

[92] Provenzano PP, Inman DR, Eliceiri KW, Knittel JG, Yan L, Rueden CT, White JG, Keely PJ (2008). Collagen density promotes mammary tumor initiation and progression. BMC Med.

[93] Finkernagel F, Reinartz S, Lieber S (2016). The transcriptional signature of human ovarian carcinoma macrophages is associated with extracellular matrix reorganization. Oncotarget.

[94] Nikitovic D, Papoutsidakis A, Karamanos NK, Tzanakakis GN (2014). Lumican affects tumor cell functions, tumor–ECM interactions, angiogenesis and inflammatory response. Matrix Biol.

[95] Cheon D-J, Tong Y, Sim M-S (2014). A collagen-remodeling gene signature regulated by TGF-β signaling is associated with metastasis and poor survival in serous ovarian cancer. Clin Cancer Res.

[96] Ames JJ, Contois L, Caron JM, Tweedie E, Yang X, Friesel R, Vary C, Brooks PC (2016). Identification of an endogenously generated cryptic collagen epitope (XL313) that may selectively regulate angiogenesis by an integrin yes-associated protein (YAP) mechano-transduction pathway. J Biol Chem.

[97] Thakur R, Mishra DP (2016). Matrix reloaded: CCN, tenascin and SIBLING group of matricellular proteins in orchestrating cancer hallmark capabilities. Pharmacol Ther.

[98] Kale S, Raja R, Thorat D, Soundararajan G, Patil TV, Kundu GC (2014). Osteopontin signaling upregulates cyclooxygenase-2 expression in tumor-associated macrophages leading to enhanced angiogenesis and melanoma growth via α9β1 integrin. Oncogene.

[99] Wilson KE, Langdon SP, Lessells AM, Miller WR (1996). Expression of the extracellular matrix protein tenascin in malignant and benign ovarian tumours. Br J Cancer.

[100] Song G, Cai Q-F, Mao Y-B, Ming Y-L, Bao S-D, Ouyang G-L (2008). Osteopontin promotes ovarian cancer progression and cell survival and increases HIF-1alpha expression through the PI3-K/Akt pathway. Cancer Sci.

[101] Song JY, Lee JK, Lee NW, Yeom BW, Kim SH, Lee KW (2009). Osteopontin expression correlates with invasiveness in cervical cancer. Aust N Z J Obstet Gynaecol.

[102] Gery S, Xie D, Yin D (2005). Ovarian carcinomas: CCN genes are aberrantly expressed and CCN1 promotes proliferation of these cells. Clin Cancer Res.

[103] Watari H, Xiong Y, Hassan MK, Sakuragi N (2009). Cyr61, a member of ccn (connective tissue growth factor/cysteine-rich 61/nephroblastoma overexpressed) family, predicts survival of patients with endometrial cancer of endometrioid subtype. Gynecol Oncol.

[104] Zhang T, Zhao C, Luo L, Xiang J, Sun Q, Cheng J, Chen D (2013). The clinical and prognostic significance of CCN3 expression in patients with cervical cancer. Adv Clin Exp Med.

[105] Kuang D-M, Wu Y, Chen N, Cheng J, Zhuang S-M, Zheng L (2007). Tumor-derived hyaluronan induces formation of immunosuppressive macrophages through transient early activation of monocytes. Blood.

[106] Kobayashi N, Miyoshi S, Mikami T (2010). Hyaluronan deficiency in tumor stroma impairs macrophage trafficking and tumor neovascularization. Cancer Res.

[107] Litwiniuk M, Krejner A, Speyrer MS, Gauto AR, Grzela T (2016). Hyaluronic acid in inflammation and tissue regeneration. Wounds.

[108] Slevin M, Krupinski J, Gaffney J, Matou S, West D, Delisser H, Savani RC, Kumar S (2007). Hyaluronan-mediated angiogenesis in vascular disease: uncovering RHAMM and CD44 receptor signaling pathways. Matrix Biol.

[109] Mambetsariev N, Mirzapoiazova T, Mambetsariev B, Sammani S, Lennon FE, Garcia JGN, Singleton PA (2010). Hyaluronic acid binding protein 2 is a novel regulator of vascular integrity. Arterioscler Thromb Vasc Biol.

[110] Singleton PA (2014). Hyaluronan regulation of endothelial barrier function in cancer. Adv Cancer Res.

[111] Bharadwaj AG, Kovar JL, Loughman E, Elowsky C, Oakley GG, Simpson MA (2009). Spontaneous metastasis of prostate cancer is promoted by excess hyaluronan synthesis and processing. Am J Pathol.

[112] Ohnuma S, Miura K, Horii A (2009). Cancer-associated splicing variants of the CDCA1 and MSMB genes expressed in cancer cell lines and surgically resected gastric cancer tissues. Surgery.

[113] Yoffou PH, Edjekouane L, Meunier L, Tremblay A, Provencher DM, Mes-Masson A-M, Carmona E (2011). Subtype specific elevated expression of hyaluronidase-1 (HYAL-1) in epithelial ovarian cancer. PLoS ONE.

[114] Nykopp TK, Rilla K, Sironen R, Tammi MI, Tammi RH, Hämäläinen K, Heikkinen A-M, Komulainen M, Kosma V-M, Anttila M (2009). Expression of hyaluronan synthases (HAS1-3) and hyaluronidases (HYAL1-2) in serous ovarian carcinomas: inverse correlation between HYAL1 and hyaluronan content. BMC Cancer.

[115] Nykopp TK, Rilla K, Tammi MI, Tammi RH, Sironen R, Hämäläinen K, Kosma V-M, Heinonen S, Anttila M (2010). Hyaluronan synthases (HAS1-3) and hyaluronidases (HYAL1-2) in the accumulation of hyaluronan in endometrioid endometrial carcinoma. BMC Cancer.

[116] Astrof S, Hynes RO (2009). Fibronectins in vascular morphogenesis. Angiogenesis.

[117] Hynes RO (2007). Cell-matrix adhesion in vascular development. J Thromb Haemost.

[118] Hielscher A, Ellis K, Qiu C, Porterfield J, Gerecht S (2016). Fibronectin deposition participates in extracellular matrix assembly and vascular morphogenesis. PLoS ONE.

[119] Zou L, Cao S, Kang N, Huebert RC, Shah VH (2012). Fibronectin induces endothelial cell migration through β1 integrin and Src-dependent phosphorylation of fibroblast growth factor receptor-1 at tyrosines 653/654 and 766. J Biol Chem.

[120] Kenny HA, Chiang C-Y, White EA (2014). Mesothelial cells promote early ovarian cancer metastasis through fibronectin secretion. J Clin Invest.

[121] Franke FE, Von Georgi R, Zygmunt M, Münstedt K (2003). Association between fibronectin expression and prognosis in ovarian carcinoma. Anticancer Res.

[122] Toh B, Wang X, Keeble J, Sim WJ, Khoo K, Wong W-C, Kato M, Prevost-Blondel A, Thiery J-P, Abastado J-P (2011). Mesenchymal transition and dissemination of cancer cells is driven by myeloid-derived suppressor cells infiltrating the primary tumor. PLoS Biol.

[123] Lawler J (2002). Thrombospondin-1 as an endogenous inhibitor of angiogenesis and tumor growth. J Cell Mol Med.

[124] Gao Q, Chen K, Gao L, Zheng Y, Yang Y-G (2016). Thrombospondin-1 signaling through CD47 inhibits cell cycle progression and induces senescence in endothelial cells. Cell Death Dis.

[125] Lawler PR, Lawler J (2012). Molecular basis for the regulation of angiogenesis by thrombospondin-1 and – 2. Cold Spring Harb Perspect Med.

[126] Martin-Manso G, Galli S, Ridnour LA, Tsokos M, Wink DA, Roberts DD (2008). Thrombospondin 1 promotes tumor macrophage recruitment and enhances tumor cell cytotoxicity of differentiated U937 cells. Cancer Res.

[127] El Rayes T, Catena R, Lee S (2015). Lung inflammation promotes metastasis through neutrophil protease-mediated degradation of Tsp-1. Proc Natl Acad Sci USA.

[128] Salvesen HB, Akslen LA (1999). Significance of tumour-associated macrophages, vascular endothelial growth factor and thrombospondin-1 expression for tumour angiogenesis and prognosis in endometrial carcinomas. Int J Cancer.

[129] Kodama J, Hashimoto I, Seki N, Hongo A, Yoshinouchi M, Okuda H, Kudo T (2001). Thrombospondin-1 and –2 messenger RNA expression in epithelial ovarian tumor. Anticancer Res.

[130] Wei W, Kong B, Qu X (2012). Alteration of HGF and TSP-1 expression in ovarian carcinoma associated with clinical features. J Obstet Gynaecol Res.

